# Regulation of Epstein-Barr Virus Minor Capsid Protein BORF1 by TRIM5α

**DOI:** 10.3390/ijms232315340

**Published:** 2022-12-05

**Authors:** Lih-Tsern Lin, Yi-Shan Lu, Hsiang-Hung Huang, Hao Chen, Shih-Wei Hsu, Li-Kwan Chang

**Affiliations:** Department of Biochemical Science and Technology, College of Life Science, National Taiwan University, Taipei City 106319, Taiwan

**Keywords:** autophagy, BORF1, Epstein-Barr virus, host-viral interaction, innate immunity, TRIM5α, TRIMosome, ubiquitination

## Abstract

TRIM5α is a host anti-retroviral restriction factor that destroys human immunodeficiency virus (HIV) virions and triggers innate immune signaling. TRIM5α also mediates the autophagic degradation of target proteins via TRIMosome formation. We previously showed that TRIM5α promotes Epstein-Barr virus (EBV) Rta ubiquitination and attenuates EBV lytic progression. In this study, we sought to elucidate whether TRIM5α can interact with and induce the degradation of EBV capsid proteins. Glutathione S-transferase (GST) pulldown and immunoprecipitation assays were conducted to identify interacting proteins, and mutants were generated to investigate key binding domains and ubiquitination sites. Results showed that TRIM5α binds directly with BORF1, an EBV capsid protein with a nuclear localization signal (NLS) that enables the transport of EBV capsid proteins into the host nucleus to facilitate capsid assembly. TRIM5α promotes BORF1 ubiquitination, which requires the surface patch region in the TRIM5α PRY/SPRY domain. TRIM5α expression also decreases the stability of BORF1(6KR), a mutant with all lysine residues mutated to arginine. However, chloroquine treatment restores the stability of BORF1(6KR), suggesting that TRIM5α destabilizes BORF1 via direct recognition of its substrate for autophagic degradation. These results reveal novel insights into the antiviral impact of TRIM5α beyond retroviruses.

## 1. Introduction

Tripartite motif 5 alpha (TRIM5α) is a species-specific restriction factor that is known to act against human immunodeficiency virus type 1 (HIV-1) infection in Rhesus monkeys and N-tropic murine leukemia virus (N-MLV) infection in humans [1,2]. TRIM5α comprises four major domains: RING, B-box, coiled-coil, and B30.2 (PRY/SPRY) [3,4,5]. The RING domain provides ubiquitin E3 ligase activity, and mutations in this region affect the ubiquitination of target proteins [6,7], thereby impacting the ability of TRIM5α to restrict viral DNA synthesis [8,9]. The B-box and coiled-coil domains are required for TRIM5α to self-associate and form higher-order structures that increase avidity with the retrovirus capsid [10,11,12]. The B30.2 (PRY/SPRY) domain is responsible for substrate recognition and binding of viral capsids [13], and the sequence of this region varies between different species; this determines the specificity of viral restriction [5].

TRIM5α promotes capsid disassembly of retroviral particles via direct binding [11,14]. Once an incoming viral particle is recognized, TRIM5α self-assembles into a hexagonal structure to trap viral capsid proteins and induce abnormal disassembly [15,16]. Moreover, TRIM5α can initiate self-K63-polyubiquitination on its N-terminus to recruit proteasomes, thereby blocking the reverse transcription of viral genomes by promoting virus destruction and triggering immune signaling [16]. Recent studies have also revealed that proteins in the TRIM family play critical roles in autophagy. During selective autophagy, polyubiquitin signals labeled on target proteins are needed to initiate the process [17]. It is also known that TRIM proteins can mediate autophagy. Through this mechanism, TRIM5α interacts with active phospho-ULK1 and BECN1 to form an autophagic platform termed TRIMosome, which is subsequently involved in the degradation of a target protein [18,19,20]. This type of autophagy is also referred to as precision autophagy since it is based on specific recognition [21]. For example, HIV p24 is recognized by TRIM5α and subsequently degraded by precision autophagy via TRIMosomes [19].

It is known that the TRIM5α not only restricts retroviruses but also represses the transposition of human retroelement LINE-1, destabilizes NS2B/3 protease of tick-borne flaviviruses, and inhibits the replication of Epstein-Barr virus (EBV) [22,23,24,25]. Our previous study revealed that TRIM5α interacts with and enhances the ubiquitination of EBV Rta, leading to its instability and reduction of transactivation activity to attenuate viral lytic progression [26]. However, little is known about the mechanisms by which TRIM5α exerts restriction against EBV. EBV capsid assembly is an important step in genome packaging and virion production [27]. The structure of the EBV capsid shell comprises a major capsid protein, VCA, two minor capsid proteins, BDLF1 and BORF1, and a small capsid protein, BFRF3 [27,28]. Of these capsid proteins, BORF1 has a nuclear localization signal (NLS) to facilitate nuclear transport [29]; the nuclear import of BDLF1 and VCA relies on their interaction with BORF1 [30]. A recent study showed that Rta is an EBV tegument protein that binds to the capsid [31]. Therefore, in this study, we wished to elucidate if TRIM5α could interact with EBV capsid proteins, possibly via the interaction with Rta, and examine how such interaction would affect EBV replication. To our knowledge, this is the first study to investigate interactions between TRIM5α and DNA virus capsid proteins. The results may have important implications for the overall role and function of TRIM5α in host antiviral defense.

## 2. Results

### 2.1. Interaction between BORF1 and TRIM5α

**Figure 1:**
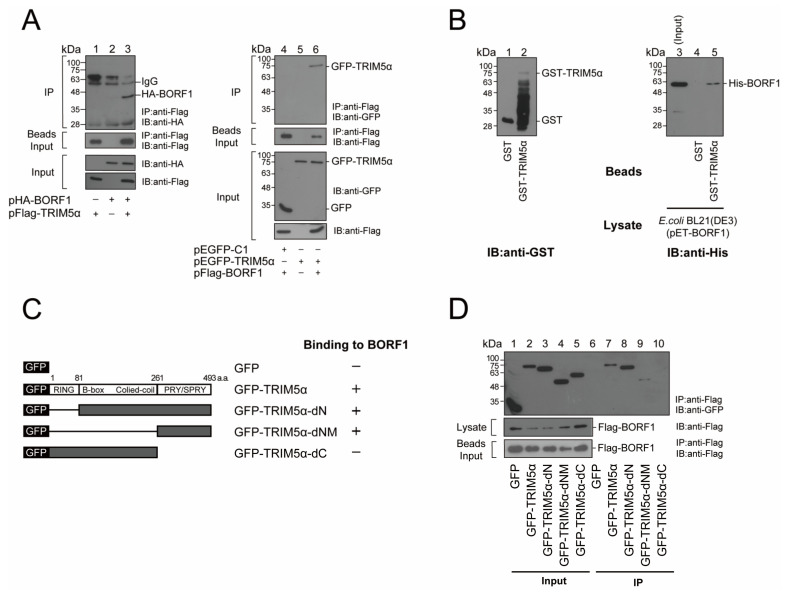
Interaction between BORF1 and TRIM5α. (**A**) HEK293T cells were transfected or cotransfected with pHA-BORF1 and pFlag-TRIM5α (lanes 1–3), or pFlag-BORF1, pEGFP-TRIM5α, and pEGFP-C1 (lanes 4–6). Proteins in the lysates were immunoprecipitated (IP) with an anti-Flag antibody, and immunoprecipitated proteins were detected by immunoblotting (IB) using an anti-HA antibody (lanes 1–3) or anti-GFP antibody (lanes 4–6). Input lanes were loaded with 3% cell lysate. (**B**) Bacterially-expressed GST (lane 1) or GST-TRIM5α (lane 2) bound to glutathione-Sepharose beads were mixed with the *E. coli* BL21(DE3)(pET-BORF1) lysate. GST and GST-fusion proteins binding to the glutathione-Sepharose beads were eluted and detected by immunoblotting with anti-GST antibody (lanes 1, 2). Proteins pulled down by the beads and His-BORF1 in 1% cell lysate (lane 3) were similarly analyzed using an anti-His antibody (lanes 4, 5). (**C**) Deletion mutants used to identify the TRIM5α regions that interact with BORF1. (**D**) HEK293T cells were cotransfected with plasmids encoding Flag-BORF1 and GFP-TRIM5α, GFP-TRIM5α-dN, GFP-TRIM5α-dNM, or GFP-TRIM5α-dC. Proteins in the cell lysates were immunoprecipitated with an anti-Flag antibody and detected by immunoblotting using an anti-GFP antibody (lanes 6–10). Input lanes were loaded with 3% cell lysate (lanes 1–5). Beads input (**A**,**D**): anti-Flag M2 beads were added to the lysates, and proteins binding to the beads were detected by immunoblotting with an anti-Flag antibody.

TRIM5α is known to interact with the capsid of HIV and induce its destruction [11,14]. Our earlier study showed that EBV Rta interacts with TRIM5α [26]. As Rta is closely associated with the EBV capsid and present in the tegument layer [31], this study, therefore, investigated whether TRIM5α could similarly interact with EBV capsid proteins. As BORF1 plays a pivotal role in bringing EBV capsid proteins into the nucleus for capsid assembly [29], this study investigated whether TRIM5α interacted with BORF1. To accomplish this, we cotransfected HEK293T cells with pHA-BORF1 and pFlag-TRIM5α, and lysates were prepared 24 h after transfection. Immunoblot analysis revealed that both HA-BORF1 and Flag-TRIM5α were detected with anti-HA and anti-Flag antibodies in the cell lysate, respectively (Figure 1A, lane 3, input). We also found that adding Flag-M2 beads to the lysate coimmunoprecipitated HA-BORF1 (Figure 1A, lane 3, IP). However, HA-BORF1 was undetected by immunoblotting if the cells were transfected with only pHA-BORF1 or pFlag-TRIM5α (Figure 1A, lanes 1, 2, IP). We also cotransfected HEK293T cells with pFlag-BORF1 and pEGFP-TRIM5α and found that GFP-TRIM5α was coimmunoprecipitated by Flag-M2 beads (Figure 1A, lane 6, IP). However, GFP-TRIM5α was undetected by immunoblotting if lysates from cells transfected with only pFlag-BORF1 or pEGFP-TRIM5α (Figure 1A, lanes 4, 5, IP) were used. These results demonstrate that BORF1 interacts with TRIM5α.

We also conducted a GST pulldown study using bacterially expressed GST, GST-TRIM5α, and His-BORF1 to verify the interaction between GST-TRIM5α and His-BORF1. Accordingly, we mixed GST-TRIM5α-glutathione-Sepharose beads or GST-glutathione-Sepharose beads with His-BORF1. The amounts of GST and GST-TRIM5α binding to the beads were analyzed by immunoblot analysis with anti-GST antibody (Figure 1B, lanes 1, 2). We found that the majority of GST-TRIM5α was degraded; only a small amount of intact GST-TRIM5α was detected (Figure 1B, lane 2). Despite the amount being small, GST-TRIM5α on the beads pulled down His-BORF1 (Figure 1B, lane 5). Some of the degraded GST-TRIM5α may still retain the binding motif and have contributed to the pulldown of BORF1. As a control, His-BORF1 was not pulled down by GST-glutathione-Sepharose beads (Figure 1B, lane 4).

**Figure 2:**
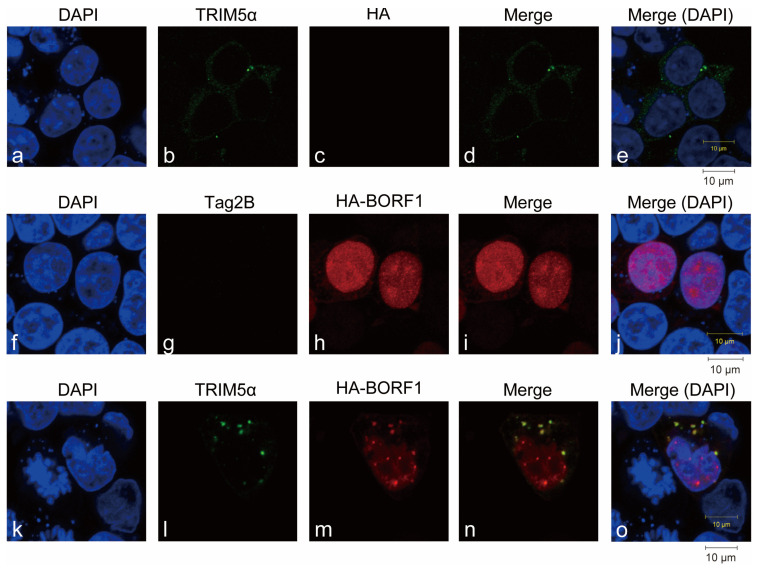
Subcellular localization of TRIM5α and BORF1. Representative confocal images of HEK293T cells expressing HA-BORF1 (**h**,**m**) and Flag-TRIM5α (**b**,**l**) or empty vector (Tag2B) (**c**,**g**) were incubated with anti-HA antibody (**c**,**h**,**m**), and anti-TRIM5α antibody (**b**,**g**,**l**). The nucleus was visualized by DAPI staining (**a,f,k**). Panel (**d**) is a merged image of (**b**,**c**). Panels (**i**) and (**n**) are merged images of (**g**,**h**) and (**l**,**m**), respectively. Panels (**e**,**j**,**o**) are merged images of (**d**,**i**,**n**) with DAPI (**a**,**f**,**k**). Scale bar: 10 μm.

This study further sought to delineate the regions in TRIM5α that interacted with BORF1. TRIM5α contains RING, B-box, coiled-coil, and B30.2(PRY/SPRY) domains. We respectively deleted these domains in TRIM5α to generate TRIM5α-dN, TRIM5α-dNM, and TRIM5α-dC, all of which were fused to GFP to facilitate detection (Figure 1C). We then cotransfected HEK293T cells with pFlag-BORF1 and a plasmid expressing GFP-TRIM5α, GFP-TRIM5α-dN, GFP-TRIM5α-dNM, or GFP-TRIM5α-dC (Figure 1C). An empty vector expressing GFP, pEGFP-C1, was used as a negative control. Immunoblotting using anti-GFP antibodies revealed the presence of GFP-TRIM5α, GFP-TRIM5α-dN, GFP-TRIM5α-dNM, GFP-TRIM5α-dC and GFP in the lysates (Figure 1D, lanes 1–5). Immunoblotting revealed that anti-Flag antibody immunoprecipitated Flag-BORF1 and coimmunoprecipitated GFP-TRIM5α, GFP-TRIM5α-dN, and GFP-TRIM5α-dNM (Figure 1D, lanes 7–9). However, GFP-TRIM5α-dC and GFP (Figure 1D, lanes 6, 10) were not coimmunoprecipitated, indicating that the B30.2(PRY/SPRY) domain (Figure 1C) in TRIM5α is key to the interaction with BORF1. Meanwhile, the localization of TRIM5α and BORF1 was examined by indirect immunofluorescence analysis. HEK293T cells were cotransfected with plasmids encoding HA-BORF1 and Flag-TRIM5α. With Flag-TRIM5α expressed alone, most of the TRIM5α were localized in the cytosol (Figure 2a–e). After the cells were cotransfected with pHA-BORF1 and pTag-2B, HA-BORF1 was localized in the nucleus (Figure 2f–j). However, with the coexpression of Flag-TRIM5α and HA-BORF1, the HA-BORF1 in the cytoplasm colocalized with Flag-TRIM5α as speckles (Figure 2k–o), showing that HA-BORF1 interacts with Flag-TRIM5α.

### 2.2. TRIM5α Is a Ubiquitin E3 Ligase of BORF1

**Figure 3:**
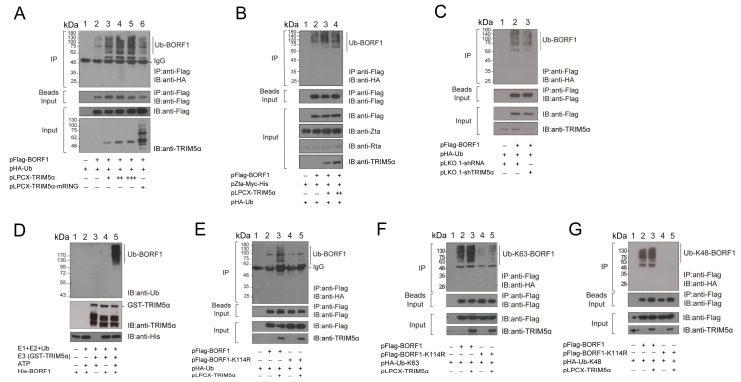
Ubiquitination of BORF1 directed by TRIM5α. (**A**,**C**,**E**–**G**) HEK293T and (**B**) 2089(Maxi-EBV) cells were transfected or cotransfected with plasmids as indicated. Proteins in the lysates were immunoprecipitated (IP) with an anti-Flag antibody, and immunoprecipitated proteins were detected by immunoblotting (IB) with an anti-HA antibody. Proteins in the lysates were also detected by IB using antibodies indicated (Input). Beads input: Immunoblotting with anti-Flag antibodies detected proteins binding to the anti-Flag M2 beads. (**D**) Ubiquitination of BORF1 in vitro. His-BORF1-Ni^2+^-NTA-Sepharose beads were added to mixtures containing bacterially expressed and purified GST-TRIM5α, bovine ubiquitin, ubiquitin E1 protein, His-UbcH5a, MgCl_2_, ATP, and GST-TRIM5α. Samples were then analyzed by immunoblotting using an anti-ubiquitin (Ub) antibody. TRIM5α and His-BORF1 in the reaction mixtures were analyzed by immunoblotting with anti-TRIM5α and anti-His antibodies, respectively.

Our earlier study showed that TRIM5α interacts and functions as a ubiquitin E3 ligase to promote the ubiquitination of EBV Rta [26]. As TRIM5α also interacted with BORF1 (Figure 1), this study further investigated whether TRIM5α promoted the ubiquitination of BORF1. We cotransfected HEK293T cells with pFlag-BORF1 and pHA-Ub. At 24 h after cotransfection, cell lysates were prepared. Proteins in the lysate were immunoprecipitated with an anti-Flag antibody. Immunoblotting of the immunoprecipitated proteins with anti-HA antibodies revealed a series of Ub-BORF1 bands (Figure 3A, lane 2, IP). These bands were unobserved if the cells were not transfected with pHA-Ub (Figure 3A, lane 1 IP), indicating that ubiquitin (Ub) chains post-translationally modify BORF1. The intensity of these Ub-BORF1 bands increased in a dose-dependent manner after cells were also cotransfected with pLCPX-TRIM5α (Figure 3A, lanes 3–5, IP), indicating that expression of TRIM5α enhances the ubiquitination of BORF1. It is known that the C15 and C18 residues in the RING domain are critical to TRIM5α’s E3 ligase activity, and mutation of these two residues subsequently abolishes E3 ligase activity [9]. Therefore, we transfected HEK293T cells with a plasmid expressing a mutant TRIM5α with C15A and C18A mutations (pLPCX-TRIM5α-mRING). The result showed that the mutant protein did not promote the ubiquitination of BORF1 as much as wild-type TRIM5α (Figure 3A, lane 6, IP), revealing the critical role of the TRIM5α RING domain in BORF1 ubiquitination. We also performed similar experiments with HEK293 cells, harboring an EBV bacmid, i.e., 2089(maxi-EBV) cells [32]. EBV in the cells was lytically activated by introducing pZta-myc-his into the cells by transfection. As expected, two immediate-early proteins, Rta and Zta, were expressed after lytic induction (Figure 3B, Input). The results revealed that overexpressing TRIM5α increased the amounts of Ub-BORF1 (Figure 3B, IP). Silencing the expression of TRIM5α with shRNA slightly reduced the ubiquitination of BORF1 (Figure 3C, lane 3, IP). To further examine whether TRIM5α is responsible for the ubiquitination of BORF1, an in vitro experiment was conducted using purified His-BORF1, ubiquitin E1 and E2 enzymes, and GST-TRIM5α. Ubiquitinated BORF1 was detected only when His-BORF1, E1, E2, GST-TRIM5α, and ATP were present in the mixture (Figure 3D, lane 5), indicating that TRIM5α is a ubiquitin E3 ligase of BORF1.

We also substituted each of the six lysine residues in BORF1 with arginine to determine which residues were conjugated by Ub. HEK293T cells were cotransfected with pHA-Ub, pLPCX-TRIM5α, and plasmids, respectively expressing Flag-BORF1 or BORF1 mutant derivatives with a lysine-to-arginine substitution. At 24 h after transfection, proteins in the lysate were immunoprecipitated with anti-Flag antibody and analyzed by immunoblotting with anti-HA antibody. We tested these six mutants and found that K114R mutation significantly reduced the ubiquitination of BORF1 (Figure 3E, lane 4, IP), regardless of whether TRIM5α was expressed (Figure 3E, lane 5, IP). This indicates that BORF1 is conjugated by Ub at K114. In addition, we used two Ub mutants with all lysine residues substituted with arginine except for K48 (K48-Ub) or K63 (K63-Ub). In a cotransfection study, we found that although BORF1 was ubiquitinated by both K63-Ub (Figure 3F, lanes 2, 3, IP) and K48-Ub (Figure 3G, lanes 2, 3, IP), ubiquitination of BORF1 by these two Ub mutants was substantially reduced after the K114R mutation (Figure 3F, lanes 4, 5, IP; Figure 3G, lanes 4, 5, IP).

### 2.3. The PRY/SPRY Domain in TRIM5α Is Critical for BORF1 Ubiquitination

**Figure 4:**
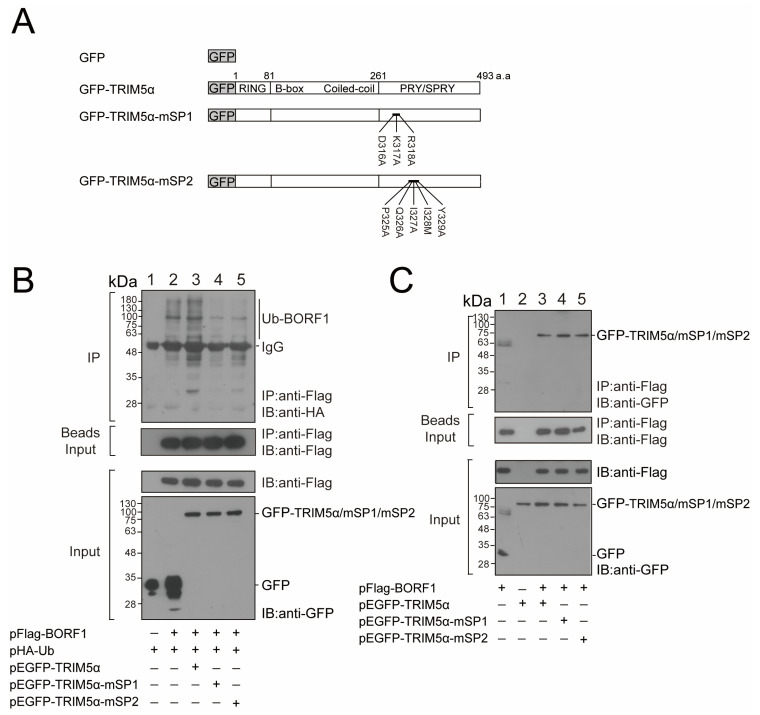
Interaction between BORF1 and the PRY/SPRY domain in TRIM5α. (**A**) Mutations generated in the PRY/SPRY domain of TRIM5α. (**B**,**C**) HEK293T cells were transfected or cotransfected with plasmids as indicated. Proteins in the cell lysates were immunoprecipitated (IP) with an anti-Flag antibody, and immunoprecipitated proteins were detected by immunoblotting (IB) with an anti-HA antibody. Proteins in the lysates were detected by IB using anti-Flag and anti-GFP antibodies (Input). Input lanes were loaded with 3% cell lysate. Proteins binding to the anti-Flag M2 beads were detected by immunoblotting with an anti-Flag antibody (Beads Input).

The PRY/SPRY domain in TRIM5α binds to the HIV capsid and plays a critical role in restricting retroviruses [33]. As we found that BORF1 interacted with this domain (Figure 1C,D), this study mutated the aa 316–318 and aa 325–329 motifs in the domain to generate TRIM5α-mSP1 and TRIM5α-mSP2 [33] (Figure 4A), respectively. HEK293T cells were cotransfected with plasmids expressing Flag-BORF1, HA-Ub, and GFP-TRIM5α, GFP-TRIM5α-mSP1, or GFP-TRIM5α-mSP2. Ub-BORF1 was immunoprecipitated with an anti-Flag antibody, followed by immunoblotting with an anti-HA antibody. The results revealed that mutations of the aa 316–318 motif in the PRY/SPRY domain reduced the ability of TRIM5α to promote the ubiquitination of BORF1 (Figure 4B, lanes 3, 4). A similar reduction in the ubiquitination of BORF1 was observed after the mutation of the aa 325–329 motif (Figure 4B, lanes 3, 5). The results indicated that the PRY/SPRY region in TRIM5α has a crucial role in promoting BORF1 ubiquitination. We also conducted a coimmunoprecipitation assay to assess the binding between the PRY/SPRY mutants and BORF1. We found that both PRY/SPRY mutants bound to Flag-BORF1 at levels comparable to wild-type TRIM5α (Figure 4C, lanes 3–5), thus confirming the lack of BORF1 ubiquitination is not due to loss of binding to TRIM5α.

### 2.4. Substituting All the Lysine Residues with Arginine in BORF1 Increases Its Stability

**Figure 5:**
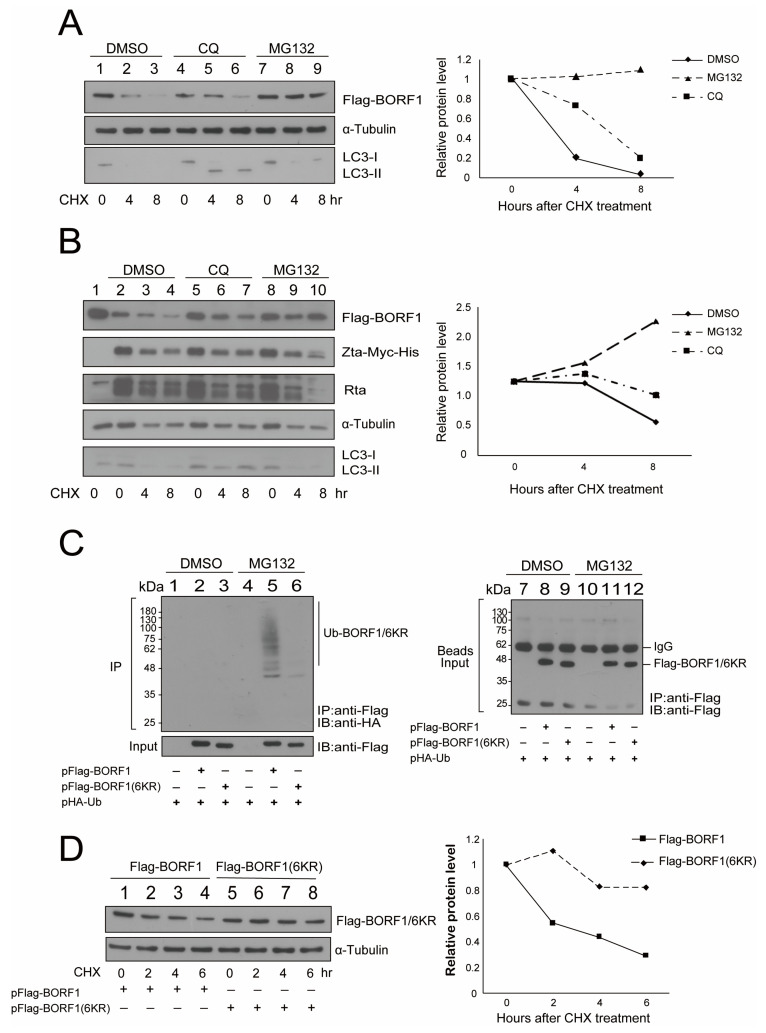
Stability of BORF1 and BORF1(6KR). (**A**) HEK293T and (**B**) 2089(Maxi-EBV) cells were transfected with pFlag-BORF1. Cotransfecting pZta-myc-His lytically activated the EBV in 2098(Maxi-EBV) cells. At 24 h after transfection, cells were treated with cycloheximide (CHX) or DMSO (lanes 1–3), CQ (lanes 4–6), or MG132 (lanes 7–9). Cells were harvested at 0, 4, or 8 h after treatment. Proteins in the lysate were analyzed by immunoblotting with the antibodies indicated. (**C**) Lysates were prepared from HEK293T cells cotransfected with plasmids expressing HA-Ub and Flag-BORF1 or Flag-BORF1(6KR). After transfection, cells were treated with DMSO (lanes 1–3) or MG132 (lanes 4–6) for 12 h. Immunoprecipitated proteins were analyzed by immunoblotting (IB) with anti-HA antibodies. Proteins in the lysates (Input) were detected with an anti-Flag antibody. Proteins binding to the beads were detected by IB using an anti-Flag antibody (Beads Input). (**D**) Cells were harvested at 0, 2, 4, or 6 h after treatment with CHX to examine the stability of Flag-BORF1 (lanes 1–4) and Flag-BORF1(6KR) (lanes 5–8). Proteins were detected by immunoblotting, and the intensity of the bands relative to that of α-tubulin was quantified using ImageJ software.

To determine how UPS and the autophagy-lysosomal pathway influenced the stability of BORF1, HEK293T cells were cotransfected with pFlag-BORF1 and treated with a protein synthesis inhibitor, cycloheximide (CHX). The cells were then treated with DMSO (control), an autophagy flux inhibitor (chloroquine, CQ), or a proteasome inhibitor (MG132). The amount of Flag-BORF1 in the cells was examined by immunoblotting at 0, 4, or 8 h after the treatment. The conversion of LC3-I to membrane-associated LC3-II was used as a marker for autophagosome formation [34]. We found that if the cells were treated with DMSO, Flag-BORF1 had a half-life of 3 h (Figure 5A, lanes 1–3); the half-life increased to 6 h if the cells were treated with CQ (Figure 5A, lanes 4–6). This study also found that Flag-BORF1 was stable after treatment of the cells with MG132 (Figure 5A, lanes 7–9). A stability increase for Flag-BORF1 was also observed in 2089(Maxi-EBV) cells after lytic induction by transfecting pZta-Myc-His (Figure 5B). Furthermore, Flag-BORF1 was more stable after MG132 treatment (Figure 5B) than with CQ (Figure 5B). This study also substituted all the lysine residues in BORF1 with arginine to create BORF1(6KR). HEK293T cells were cotransfected with pHA-Ub and pFlag-BORF1 or pFlag-BORF1(6KR). Immunoprecipitation verified that BORF1(6KR) was not modified by ubiquitin (Figure 5C, lane 6). We treated the cells with cycloheximide and found that BORF1(6KR) (Figure 5D, lanes 5–8) was more stable than BORF1 (Figure 5D, lanes 1–4), likely due to the lack of ubiquitination.

### 2.5. TRIM5α Destabilizes BORF1(6KR) via Autophagy

**Figure 6:**
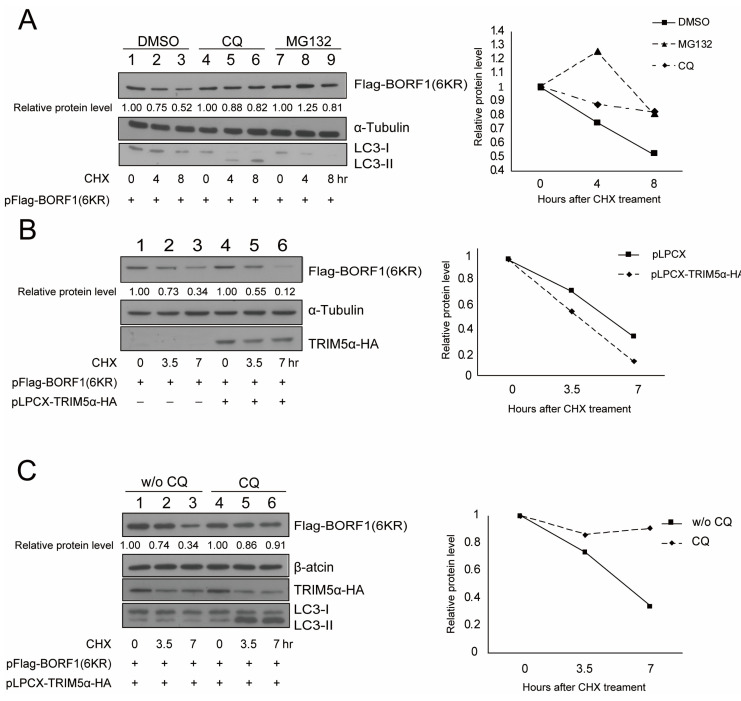
Influence of TRIM5α and chloroquine (CQ) on the stability of BORF1(6KR). (**A**) HEK293T cells were transfected with pFlag-BORF1(6KR). At 24 h after transfection, cells were treated with cycloheximide (CHX) and CQ (lanes 4–6), MG132 (lanes 7–9), or DMSO (lanes 1–3). Cells were harvested at 0, 4, or 8 h after treatment. Proteins in the lysate were analyzed by immunoblotting with the antibodies indicated. (**B**) HEK293T cells were cotransfected with pFlag-BORF1(6KR) and pLPCX-TRIM5α-HA (lanes 4–6) or transfected with pFlag-BORF1(6KR) (lanes 1–3). At 24 h after transfection, cells were treated with CHX. Cells were harvested at 0, 3.5, and 7 h after treatment. (**C**) A similar experiment was performed, except that the cells were treated (lanes 4–6) or untreated (lanes 1–3) with CQ. Proteins in the lysate were detected by immunoblotting with antibodies indicated; expression levels relative to α-tubulin or β-actin were quantified using ImageJ software.

We examined the stability of BORF1(6KR) after transfection of HEK293T cells and by treating the cells with CQ or MG132. The results showed that, compared with the levels of BORF1(6KR) in cells treated with DMSO (Figure 6A, lanes 1–3), both CQ and MG132 treatments increased the stability of BORF1(6KR) (Figure 6A, lanes 4–9). The stabilization of BORF1(6KR) by MG132 and CQ, respectively, suggested that BORF1(6KR) is degraded by proteasome via a ubiquitination-independent pathway or directed to the lysosomal degradation pathway. As BORF1 interacts with TRIM5α, we sought to ascertain whether BORF1 was degraded by TRIM5α-mediated precision autophagy. We found that the stability of Flag-BORF1(6KR) decreased by 66% at 7 h after transfection (Figure 6B). However, stability decreased by 88% if the cells were cotransfected with pLPCX-TRIM5α-HA (Figure 6B), suggesting that the expression of TRIM5α decreased the stability of BORF1(6KR). To determine if TRIM5α directs BORF1 degradation through an autophagy-lysosomal pathway, we examined the stability of BORF1(6KR) by treating the cells with CQ after overexpressing TRIM5α. BORF(6KR) was stabilized after the treatment (Figure 6C, lanes 4–6). These results suggested that TRIM5α directs BORF1(6KR) to autophagy degradation, and CQ prevents the fusion of the autophagosome and lysosome, thus increasing the stability of BORF1(6KR).

### 2.6. BORF1(6KR) Interacts with TRIM5α, p62, and BECN1

**Figure 7:**
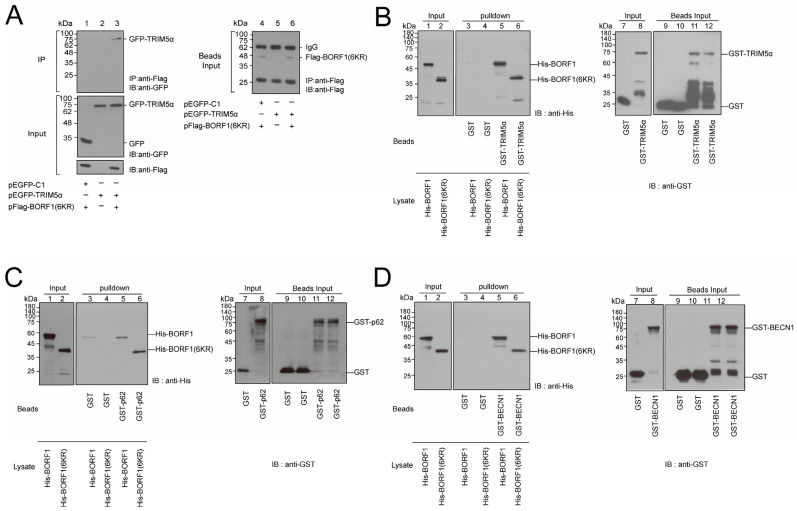
Interaction between BORF1(6KR) and TRIM5α, p62, and BECN1. (**A**) HEK293T cells were transfected or cotransfected with pEGFP-C1, pEGFP-TRIM5α, and pFlag-BORF1(6KR) as indicated. Proteins in the cell lysate were immunoprecipitated (IP) with an anti-Flag antibody, followed by immunoblotting (IB) with an anti-GFP antibody. Flag-BORF1 and GFP-TRIM5α in the lysates (Input) and Flag-BORF1 on the anti-Flag M2 beads (Beads Input) were examined by immunoblotting as shown. (**B**) GST (lane 7) and GST-TRIM5α (lane 8) were bound to glutathione-Sepharose beads. The beads were respectively added to the *E. coli* BL21(DE3)(pET32a-BORF1) (lanes 1, 3, 5) and *E. coli* BL21(DE3)[pET28a-BORF1(6KR)] (lanes 2, 4, 6) lysates. Proteins binding to the beads were analyzed by IB with anti-His antibody (lanes 3–6). Input lanes were loaded with 1% cell lysate. Beads input for lanes 3–6 is respectively shown in lanes 9–12. The pulldown of His-BORF1 and His-BORF1(6KR) by GST-p62 and GST-BECN1 was similarly presented in (**C**) and (**D**), respectively.

HEK293T cells were transfected or cotransfected with pEGFP-TRIM5α and pFlag-BORF1(6KR); proteins in the lysate were coimmunoprecipitated with anti-Flag antibody and detected by immunoblotting with anti-GFP antibody. The results showed that GFP-TRIM5α was coimmunoprecipitated with Flag-BORF1(6KR) by an anti-Flag antibody (Figure 7A, lane 3, IP). However, GFP-TRIM5α was not coimmunoprecipitated if the cells were transfected with pEGFP-TRIM5α alone (Figure 7A, lane 2, IP), demonstrating that Flag-BORF1(6KR) interacts with GFP-TRIM5α in vivo. The GST-pulldown assay also showed that GST-TRIM5α-glutathione-Sepharose beads pulled down bacterially-expressed His-BORF1 and His-BORF1(6KR) (Figure 7B, lanes 5, 6). However, His-BORF1 and His-BORF1(6KR) were not pulled down by GST-glutathione Sepharose beads (Figure 7B, lanes 3, 4). The pulldown results confirmed a direct binding between TRIM5α and BORF1(6KR) and suggested that the 6KR mutation does not affect the binding of BORF1 to TRIM5α. This study also examined if His-BORF1 and His-BORF1(6KR) are bound to the components of the TRIMosome, including p62 and BECN1. The results showed that GST-p62-glutathione Sepharose beads pulled down both His-BORF1 and His-BORF1(6KR) from the *E. coli* BL21(DE3)(pET-BORF1) or *E. coli* BL21(DE3)[pET-BORF1(6KR)] lysate, respectively (Figure 7C, lanes 5, 6). A similar pulldown experiment using GST-BECN1 showed that His-BORF1(6KR) was pulled down by GST-BECN1-glutathione-Sepharose beads (Figure 7D, lane 6) but not by GST-glutathione-Sepharose beads (Figure 7D, lane 4), indicating that His-BORF1 and His-BORF1(6KR) interact with GST-p62 and GST-BECN1 directly in vitro. Notably, although the His-BORF1(6KR) band detected by immunoblotting migrated to a position consistent with its molecular mass, His-BORF1 migrated slower than His-BORF1(6KR) (Figure 7D, lanes 1, 2, 5, and 6). This was because His-BORF1 was expressed from a plasmid with a pET-32a backbone and contained a fusion of an extra 109-amino acid Trx tag.

## 3. Discussion

The TRIM5α restriction factor is known to restrict HIV-1 infection in a species-specific manner [1,2,15,16]. TRIM5α forms high-order hexagonal assemblies on incoming retroviral capsids, thus interfering viral life cycle or triggering antiviral responses [35]. Recently, structurally diverse substrates, including human retroelement LINE-1 and tick-borne flavivirus, have been discovered to be restricted by TRIM5α, suggesting TRIM5α acts as an antiviral factor with a broad spectrum [25]. Several studies indicated that TRIM5α mediates precision autophagy via the TRIMosome complex, thereby promoting degradation of its bound cargo, such as the HIV p24 protein [18,19]. Other than a retrovirus, TRIM5α is a ubiquitin E3 ligase of Rta and attenuates the EBV lytic cycle by affecting the abundance of Rta, a transcription factor required for lytic progression [26]. Here, we demonstrated that TRIM5α is a ubiquitin E3 ligase of an EBV capsid protein, BORF1, and the surface patch region of TRIM5α contributes to the E3 ligase activity. During EBV lytic progression, BORF1, with its NLS, is responsible for transporting other capsid proteins into the nucleus to complete the capsid assembly process [29,30]. This study demonstrates that TRIM5α causes BORF1 degradation by mediating selective autophagy via direct binding, revealing a novel TRIM5α restriction strategy that targets the capsid protein of a DNA virus.

This study identified an interaction between TRIM5α and BORF1, demonstrated by coimmunoprecipitation, GST pull-down assay, and indirect immunofluorescence analysis (Figure 1 and Figure 2). TRIM5α interacts with BORF1 via its PRY/SPRY domain (Figure 1), which is also required for recognizing the HIV-1 capsid [24]. The results suggest that TRIM5α utilizes this domain to recognize its cargo. Additionally, our earlier study revealed that TRIM5α is mainly localized in the cytosol during latency but colocalizes with Rta in the nucleus after lytic activation [26]. Rta is a tegument protein that colocalizes with BORF1 in the nucleus during lytic induction [31], suggesting that TRIM5α is involved in nucleocapsid assembly. Whether TRIM5α recognizes EBV capsids during lytic activation will be further elucidated.

This study demonstrates that TRIM5α promotes BORF1 ubiquitination both in vivo and in vitro (Figure 3). Mutation of the RING domain in TRIM5α or knockdown of the expression of TRIM5α reduces levels of ubiquitinated BORF1 (Figure 3A,C). Yet, the reduction seems not to be dramatic. It is possible that TRIM5α is not the only ubiquitin E3 ligase of BORF1. So far, two viral proteins, flaviviral protease NS2B/3 and EBV Rta, have been reported to be the substrates of TRIM5α-mediated ubiquitination [23,26]. This is the first case showing that TRIM5α influences the stability and proliferation of a DNA virus. To analyze the lysine residue in BORF1 conjugated by ubiquitin, this study generated BORF1 mutants with lysine-to-arginine substitutions. We generated single or double lysine mutants of BORF1 for the ubiquitination assay. Among the mutations, the K114R substitution significantly reduces the ubiquitination by wild-type Ub (Figure 3E), Ub mutants with all the K residues substituted by R except for K63 (Figure 3F) or K48 (Figure 3G), suggesting that K63 and K48 ubiquitination chains at K114 in BORF1 conjugate BORF1. Although the signal of ubiquitinated BORF1 that is conjugated by the K63 chain (Figure 3F) seems more intense than that conjugated by the K48 chain (Figure 3G), we cannot conclude that a K63-polyubiquitination chain predominantly conjugates BORF1 as the results were from independent experiments with a different exposure time of the blots. We also verified the two surface patch areas, SP1 and SP2, in the B30.2(PRY/SPRY) domain contribute to the ubiquitin E3 ligase activity of TRIM5α (Figure 4). The mutation of these residues in Rhesus monkey TRIM5α decreases the restriction potency against HIV-1 without affecting the binding activity to the HIV capsid [33]. Our findings suggest that the surface patch areas of TRIM5α participate in the viral restriction process via E3 ligase activity contribution.

This study found that BORF1 stability is regulated by autophagy and proteasome degradation but primarily mediated by proteasomal degradation (Figure 5A). Recent studies have shown reciprocal cross-talks between proteasome and autophagic pathways [36,37], such as proteasome inhibition upregulating autophagic degradation [38]. We thus generated a BORF1 mutant with all the lysine residues replaced by arginine, BORF1(6KR), to mitigate the interference of ubiquitin modification signaling. We subsequently verified that BORF1(6KR) is not ubiquitinated (Figure 5C) but still shows direct interaction with TRIM5α (Figure 7A,B). Importantly, treatments with both MG132 or CQ lead to the accumulation of BORF1(6KR) (Figure 6A). The half-life of BORF1(6KR) was longer than that of BORF1 (Figure 5D). When overexpressing TRIM5α, BORF1(6KR) became unstable (Figure 6B), but stability was restored if cells were treated with CQ (Figure 6C). Previous studies indicated that TRIM5α acts as a scaffolding platform for autophagy by direct binding to its cargo [19,21]. We found that TRIM5α binds to BORF1(6KR) directly (Figure 7B) and further demonstrated that BORF1(6KR) binds to p62 and BECN-1 directly (Figure 7C,D). The results support that TRIM5α recognizes BORF1(6KR) and forms a TRIMosome for precision autophagy.

Capsid assembly of EBV takes place in the host cell nucleus, and the entry of major capsid protein VCA and a minor capsid protein BDLF1 relies on the nuclear translocation ability of BORF1 [29]. Our previous study showed that knocking down TRIM5α in P3HR1 cells, which increases the stability of Rta, promotes lytic protein expression and the production of virions [26]. This study further verifies that TRIM5α destabilizes not only Rta but also BORF1 and indicates that TRIM5α participates in the destabilization of EBV capsid proteins via a novel mechanism of precision autophagy. Further research is warranted to examine this impact on EBV viability and propagation by using cells containing recombinant EBV with BORF1(6KR).

## 4. Materials and Methods

### 4.1. Cell Line

HEK293T cells were isolated from embryo kidney epithelium [39]. They were cultured in Dulbecco’s modified Eagle’s medium (DMEM) containing 10% fetal calf serum, 100 µg/mL penicillin G, 100 µg/mL streptomycin, and 2 mM L-glutamine at 37 °C with 5% CO_2_. 2089(Maxi-EBV) cells, 293 cells harboring an EBV bacmid [32], were cultured in DMEM supplemented with 10% fetal calf serum.

### 4.2. Plasmids

Plasmids including GST, GST-TRIM5α, pLPCX-TRIM5α-HA, pLPCX-TRIM5α, pEGFP-TRIM5α, pEGFP-TRIM5α-dN, pEGFP-TRIM5α-dNM, pEGFP-TRIM5α-dC, pHA-Ub, pcDNA-BORF1, pET-BORF1, and pFlag-BORF1 have been described earlier [26]. Plasmid pFlag-BORF1-K114R was constructed by site-directed mutagenesis using pFlag-BORF1 as a template and amplified by PCR using primers BORF1-K114R-F (5’-TCCAATTCAGGCAGAGTGAC) and BORF1-K114R-R (5’-GTCACTCTGCCTGAATTGGA). The original template was then digested by restriction enzyme DpnI, and the PCR product was transformed into *Escherichia coli* EPI300. Plasmid pFlag-BORF1-6KR was constructed with the same method as pFlag-BORF1-K114R, but used pFlag-BORF1-K2R, K196R, K202R, K214R, K222R (Tables S1 and S2) as PCR template. Plasmid pET-BORF1(6KR) was constructed by inserting the BORF1-6KR fragment, pFlag-BORF1(6KR) digested with BamHI and EcoRI restriction enzymes, into the BamHI and EcoRI sites of the pET28a vector. For pGEX-BECN1 plasmid construction, PCR fragments were amplified by primers Beclin1-F-BamHI (5’-CTTAGGATCCATGGAAGGGTCTAAGACGTCCAAC) and Beclin1-R-SalI (5’-GGCGGTCGACTCATTTGTTATAAAATTGTGAGGACACCC), using cDNA from MCF7 cells as a template, and then inserted into the BamHI and SalI sites of vector pGEX-4T1 (GE Healthcare, Chicago, Illinois, USA). Plasmid pGEX-p62 was constructed by inserting the p62 PCR segment, amplified by primers p62-F-EcoRIb(5’-CCGGGAATTCAATGGCGTCGCTCACCGTGAAG) and p62-R-XhoI (5’-CCGCTCGAGTTTCACAACGGCGGGGGATG) using cDNA from MCF7 cells as a template, into the EcoRI and XhoI sites of pGEX-4T3 (GE Healthcare, Chicago, Illinois, USA). Plasmid pFlag-TRIM5α was constructed by inserting the TRIM5α DNA fragment, amplified by PCR using pLPCX-TRIM5α as a template and primers EcoRI-TRIM5α-F (5′-CCGGAATTCATGGCTTCTGGAATCCTGGT) and SalI-TRIM5α-R (5′-ACGCGTCGACTCAAGAGCTTGGTGAGCACA), into the EcoRI and SalI sites of pCMV-Tag2B (Agilent Technologies, Santa Clara, California, USA). The plasmid expressing the TRIM5α RING mutant containing two point mutations, C15A and C18A, was constructed by inserting the corresponding PCR fragment into the EcoRI and SalI sites in pLPCX- TRIM5α. For plasmid construction of pEGFP-TRIM5α-mSP1 (which expresses TRIM5α with D316A, K317A, and R318A mutations), pEGFP-TRIM5α was used as the PCR template, and the DNA segment was first amplified using primers EcoRI-TRIM5α-F and TRIM5α-mSP1-R (5′-GCTCACTTGTGCCGCAGCTTCAGAAATGAC), as well as TRIM5α-mSP1-F (5′-GTCATTTCTGAAGCTGCGGCACAAGTGAGC) and SalI-TRIM5α-R. The amplified segments were then ligated together and elongated using primers EcoRI-TRIM5α-F and SalI-TRIM5α-R and were subsequently inserted into the EcoRI and SalI site of the pEGFP-C2 vector. Plasmid pEGFP-TRIM5α-mSP2 (which expresses TRIM5α with P325A, Q326A, I327A, I328M, and Y329A mutations) was constructed using the same method but used primers TRIM5α-mSP2-R (5′-CGTGCCCCAGCCATTGCCGCTGCTTTCGGAG) and TRIM5α-mSP2-F (5′-CTCCGAAAGCAGCGGCAATGGCTGGGGCACG) instead of TRIM5α-mSP1-R and TRIM5α-mSP1-F. Plasmids pHA-Ub-K63 and pHA-Ub-K48 express ubiquitin with whole lysine mutation, except for the K63 residue or K48 residue, respectively (Table S1). Plasmids pLKO.1-shRNA and pLKO.1-shTRIM5αwere purchased from the National RNAi Core Facility, Genomic Research Center, Academia Sinica, Taipei, Taiwan. Plasmid pLKO.1-shRNA expressing scrambled short hairpin RNA (target sequence: 5′-CCTAAGGTTAAGTCGCCCTCG-3′) served as a knockdown control. Plasmid pLKO.1-shTRIM5α, which expresses TRIM5α shRNA (target sequence: 5′-CCAGACATTTGTGAATTTCAA-3′), was used to knockdown TRIM5α in HEK293T cells. Plasmid pZta-Myc-His was constructed by inserting a Zta DNA fragment, amplified by using pFlag-Zta as a template and primers Zta-EcoRI-F (5′- CGCGAATTCATGATGGACCCAAACTCGACTTCT) and Zta-XhoI-R (5′- GGCCTCGAGGCGAAATTTAAGAGATCCTCGTGTAAAACATCTGGT), into the EcoRI and XhoI sites of pcDNA3.1-Myc-His.

### 4.3. Transient Transfection

HEK293T cells were transfected with the plasmids indicated in each experiment using Turbofect (Thermo Fisher Scientific, Waltham, Massachusetts, USA) according to the instructions provided by the manufacturer [26,40,41]. At 24–48 h post-transfection, cells were harvested and then lysed using the buffers described in the following sections.

### 4.4. Protein Production

Target plasmids were transformed into *E. coli* BL21(DE3), and the bacterial broth was cultured overnight in a 37 °C incubator. Cultures were then amplified in fresh LB broth with 25× volume and cultured in a 37 °C incubator until the optical density 600 (OD600) reached 0.5–0.6. After adding 0.2 mM isopropyl- β-D-thiogalactoside (IPTG), bacteria were cultured at 30 °C for 4–5 h to induce protein expression.

### 4.5. Western Blot Analysis

Protein samples were mixed with sample buffer (62.5 mM Tris-HCl pH 6.8, 2% sodium dodecyl sulfate (SDS), 5% β-mercaptoethanol, 10% glycerol, 2 mM ethylenediaminetetraacetic acid (EDTA), 0.005% bromophenol blue). After heating at 95 °C for 5 min, proteins were then separated by 10–12% SDS-polyacrylamide gel electrophoresis (SDS-PAGE) gel at 168V and electro-transferred to nitrocellulose (NC) membrane (Merck Millipore, Burlington, MA, USA) in transfer buffer (25 mM Tris-HCl, 5.5% glycine, 20% methanol) at 94V, 400 mA, for 1 h. After blocking with 5% defatted milk (Anchor, Auckland, New Zealand) in TBST buffer (50 mM Tris-HCl pH 7.5, 150 mM NaCl, 0.05% Tween 20) for 20 min, membranes were then probed by anti-TRIM5α (Santa Cruz, Dallas, TX, USA), anti-Flag (Sigma, St. Louis, MO, USA), anti-BORF1 [31], anti-HA (Roche, Basel, Switzerland, Cell Signaling, Danvers, MA, USA), anti-GFP (Santa Cruz, Dallas, TX, USA), anti-His (Sigma), anti-GST (Santa Cruz, Dallas, TX, USA), anti-LC3B (GeneTex, Irvine, CA, USA), anti-Ub (Santa Cruz, Dallas, TX, USA), and anti-α-tubulin (Sigma, St. Louis, MO, USA) antibodies.

### 4.6. Coimmunoprecipitation Assay

HEK293T cells at 24 h post-transfection were collected by centrifugation, then lysed using mRIPA buffer [50 mM Tris-HCl, pH 7.8, 150 mM NaCl, 5 mM EDTA, 0.5% Triton X-100, 0.5% IGEPAL, 10 μg/mL leupeptin, 10 μg/mL 4-(2-aminoethyl)benzenesulfonyl fluoride hydrochloride (AEBSF)]. Target proteins in the lysate were immunoprecipitated by mixing with 25 µL anti-Flag M2 beads (Sigma, St. Louis, MO, USA) at 4 °C for 1 h, after which beads were washed with mRIPA buffer three times 30 µL of sample buffer was added for elution. Samples were then analyzed by western blotting.

### 4.7. GST Pull-Down Analysis

*E. coli* BL21(DE3) expressing GST-tagged target proteins were harvested, then lysed using bacteria lysing buffer (0.5% IGEPAL in phosphate-buffered saline (PBS) with 1 mg/mL lysozyme, 10 μg/mL leupeptin, and 10 μg/mL AEBSF). After centrifuging at 13,800× *g* for 5 min, the supernatant was collected and mixed with 30 µL Glutathione Sepharose 4B beads (GE Healthcare, Chicago, IL, USA) at 4 °C for 1 h. Beads were then washed with 0.3% IGEPAL in PBS once. *E. coli* BL21(DE3) expressing His-tagged target proteins were also lysed using the same method and were mixed with 30 µL Glutathione Sepharose 4B beads (GE Healthcare, Chicago, IL, USA) at 4 °C for 1 h, then washed by 0.3% IGEPAL in PBS three times. After washing, 30 µL sample buffer was added for elution, and samples were analyzed by western blotting.

### 4.8. Immunoprecipitation under Denaturing Conditions

HEK293T cells at 24 h post-transfection were collected using 10 mM N-ethylmaleimide (NEM) (Sigma, St. Louis, MO, USA) in PBS to inhibit de-SUMOylation. After centrifugation, cells were lysed using denaturing lysis buffer (Solution I: 5% SDS, 150 mM Tris-HCl pH 6.7, 30% glycerol; Solution II: 25 mM Tris-HCl, pH 8.2, 50 mM NaCl, 0.5% IGEPAL, 0.1% sodium azide, 0.1% SDS; Solutions I and II were mixed in a volume ratio of 1:3, and then 5 mM DTT, 10 μg/mL leupeptin, and 10 μg/mL AEBSF were added) at 95 °C for 20 min to destroy protein-protein interactions [26,40,41]. Target proteins in the lysate were then immunoprecipitated by mixing with 25 µL anti-Flag M2 beads (Sigma, St. Louis, MO, USA) at 4 °C for 90 min, after which beads were washed with 0.5% IGEPAL in PBS three times, and 30 µL sample buffer were added for elution. Samples were then analyzed by western blotting.

### 4.9. In Vitro Ubiquitination Assay

*E. coli* BL21(DE3) expressing GST-TRIM5α or His-BORF1 were lysed with 0.2% Triton X-100 in PBS, and the lysate was centrifuged at 13,800× *g* for 5 min. The supernatant containing GST-TRIM5α or His-BORF1 was respectively mixed with 30 µL Glutathione Sepharose 4B beads (GE Healthcare, Chicago, IL, USA) or 30 μL Ni^2+^-NTA-Sepharose at 4 °C for 1 h. After washing once with 0.2% Triton X-100 in PBS, GST-TRIM5α was eluted with glutathione elution buffer (50 mM Tris-HCl, 10 mM glutathione). For ubiquitination reactions, 4.8 μg bovine ubiquitin (Sigma, St. Louis, MO, USA), 56 ng ubiquitin E1 protein (Boston Biochem, Minneapolis, MN, USA), 160 ng His-UbcH5a, 5 mM MgCl_2_, 2 mM ATP, GST-TRIM5α, and the His-BORF1 beads mixture were mixed and allowed to react at 32 °C for 90 min [41]. Samples were then analyzed by western blotting.

### 4.10. Protein Stability Analysis

HEK293T cells transfected with target plasmids were treated with 65 μg/mL cycloheximide (Sigma, St. Louis, MO, USA), combined with treatments of 50 μM CQ or 15 μM MG132. Cells were harvested at different time points and were lysed with RIPA buffer (50 mM Tris-HCl, pH 7.8, 150 mM NaCl, 5 mM EDTA, 0.1% SDS, 1% IGEPAL, 10 μg/mL leupeptin, 10 μg/mL AEBSF). Signals of target proteins were detected by western blotting and were analyzed by ImageJ.

### 4.11. Immunofluorescence Analysis

HEK293T cells were cotransfected with pHA-BORF1 and pFlag-TRIM5α. After culturing for 24 h, cells were fixed with 4% paraformaldehyde in PBS for 30 min, as described earlier [41]. Immunostaining was conducted using rabbit anti-HA monoclonal antibody (Roche, Basel, Switzerland, Cell Signaling, Danvers, MA, USA) and mouse anti-TRIM5α monoclonal antibody (Santa Cruz, Dallas, TX, USA). Cells were then treated with Alexa Fluro 594-conjugated goat anti-rabbit IgG polyclonal antibody (Invitrogen, Waltham, MA, USA) and Alexa Fluro 647-conjugated goat anti-mouse IgG polyclonal antibody (Invitrogen, Waltham, MA, USA). Nuclei were visualized by staining with 5 μg/mL 4′-6-diamidino-2-phenylindole (DAPI). Finally, cells were observed under a Zeiss confocal laser-scanning microscope (Leica TCSSP8).

## 5. Conclusions

TRIM5α is a well-known host restriction factor of retrovirus infection, but its role in infections involving other types of viruses remains unclear. We previously showed that TRIM5α attenuates the lytic cycle of EBV, showing that TRIM5α influences the proliferation of a DNA virus [26]. In this study, we demonstrate that TRIM5α acts as an E3 ligase of an EBV minor capsid protein, BORF1, via direct binding. The surface patch area of TRIM5α identified in a previous study [33] was shown to be involved in the ubiquitination function of TRIM5α. Through stability analysis of a BORF1(6KR) mutant, we find that BORF1 is a TRIMosome substrate in TRIM5α-mediated precision autophagy. As BORF1 is the only EBV capsid protein shown to have an NLS, the destabilization of BORF1 may disrupt the nuclear transportation of EBV capsid proteins and cause defects in capsid assembly. To the best of our knowledge, this is the first evidence that TRIM5α targets the capsids of a DNA virus; moreover, this study presents the first example of viral restriction involving TRIM5α-mediated precision autophagy. Further research into this mechanism is warranted.

## Data Availability

The data presented in this study are available on request from the corresponding author.

## Supplementary Materials

The following supporting information can be downloaded at: https://www.mdpi.com/article/10.3390/ijms232315340/s1.
