# Supplementary material for: Regulation of Epstein-Barr Virus Minor Capsid Protein BORF1 by TRIM5α
Source: Int J Mol Sci. 2022 Dec 5;23(23):15340. doi: 10.3390/ijms232315340 (PMC9735550; doi:10.3390/ijms232315340)
Supplement: Supplementary file 1 [file ijms-23-15340-s001.zip › ijms-1736583-SI.pdf]

International Journal of
Molecular Sciences

Supplementary Materials

Table S1. Plasmid construction methods.

Plasmid	Construct Method
**pFlag-BORF1-K196R, K202R**	Expresses Flag-tagged BORF1 protein with K196R and K202R mutations. BORF1-K196R, K202R DNA segment was first amplified by PCR using primers BamHI-BORF1-F, BORF1-K196RK202R-R and BORF1-K196RK202R-F, XhoI-BORF1-R, with pFlag-BORF1 as a template. PCR products were then ligated and elongated using primers BamHI-BORF1-F and XhoI-BORF1-R, and inserted into the BamHI and XhoI sites of pCMV-Tag2B (Agilent Technologies).
**pFlag-BORF1-K2R, K196R, K202R**	Expresses Flag-tagged BORF1 protein with K2R, K196R, and K202R mutations. BORF1-K2R, K196R, K202R DNA segment was amplified by PCR using primers BamHI-BORF1-K2R-F and XhoI-BORF1-R, with pFlag-BORF1-K196RK202R as a template, then inserted into the BamHI and XhoI sites of pCMV-Tag2B (Agilent Technologies).
**pFlag-BORF1-K2R, K196R, K202R, K214R, K222R**	Expresses Flag-tagged BORF1 protein with K2R, K196R, K202R, K214R and K222R mutations. BORF1- K2R, K196R, K202R, K214R, K222R DNA segment was first amplified by PCR using primers BamHI-BORF1-F, BORF1-K214RK222R-R and BORF1-K214RK222R-F, XhoI-BORF1-R, with pFlag-BORF1-K2R, K196R, K202R as a template. PCR products were then ligated and elongated using primers BamHI-BORF1-F and XhoI-BORF1-R, and inserted into the BamHI and XhoI sites of pCMV-Tag2B (Agilent Technologies).
**pFlag-Ub-K48R**	Expresses Flag-tagged ubiquitin protein with K48R mutation. Ub-K48R DNA segment was first amplified by PCR using primers Ub-F-EcoRI, Ub-K48R-R and Ub-R-HindIII, Ub-K48R-F, with pFlag-Ub [37] as a template. PCR products were then ligated and elongated using primers Ub-F-EcoRI and Ub-R-HindIII, and inserted into the EcoRI and HindIII sites of pCMV-Tag2B (Agilent Technologies).
**pFlag-Ub-K48RK63R**	Expresses Flag-tagged ubiquitin protein with K48R and K63R mutations. Ub-K48RK63R DNA segment was amplified by PCR using primers Ub-F-EcoRI and UbK63R-R-HindIII, with pFlag-Ub as a template. PCR products were then inserted into the EcoRI and HindIII sites of pCMV-Tag2B (Agilent Technologies).
**pFlag-Ub-6KR (K6R, K11R, K27R, K29R, K48R, K48R)**	Expresses Flag-tagged ubiquitin protein with 6 lysine mutations (K6R, K11R, K27R, K29R, K48R, K48R). Ub-6KR DNA segment was first amplified by PCR using primers UbK6RK11R-F-EcoRI, UbK27RK29R-R and UbK27RK29R-F, UbK63R-R-HindIII, with pFlag-Ub-K48RK63R as a template. PCR products were then ligated and elongated by primers UbK6RK11R-F-EcoRI and UbK63R-R-HindIII, and inserted into the EcoRI and HindIII sites of pCMV-Tag2B (Agilent Technologies).
**pFlag-Ub△K**	Expresses Flag-tagged ubiquitin protein with all 7 lysine mutations (K6R, K11R, K27R, K29R, K33R, K48R, K48R). Ub△K DNA segment was first amplified by PCR using primers UbK6RK11R-F-EcoRI, UbK33R-R and UbK33R-F, UbK63R-R-HindIII, with pFlag-Ub-6KR as a template. PCR products were then ligated and elongated by primers UbK6RK11R-F-EcoRI and UbK63R-R-HindIII, and inserted into the EcoRI and HindIII sites of pCMV-Tag2B (Agilent Technologies).
**pFlag-Ub-K48**	Expresses Flag-tagged ubiquitin protein with all lysine mutations except K48. Ub-K48 DNA segment was first amplified by PCR using primers UbK6RK11R-F-EcoRI, UbK48-R and UbK48-F, UbK63R-R-HindIII, with pFlag-Ub△K as a template. PCR products were then ligated and elongated by primers UbK6RK11R-F-EcoRI and UbK63R-R-HindIII, and inserted into the EcoRI and HindIII sites of pCMV-Tag2B (Agilent Technologies).
**pFlag-Ub-K63**	Expresses Flag-tagged ubiquitin protein with all lysine mutations except K63. Ub-K63 DNA segment was amplified by PCR using primers UbK6RK11R-F-EcoRI and UbK63R-HindIII, with pFlag-Ub△K as a template. PCR products were then inserted into the EcoRI and HindIII sites of pCMV-Tag2B (Agilent Technologies).

Table S2. Primers used in the construction of plasmids in Table S1.

Primer	Sequence
**BamHI-BORF1-F**	5’-CGCGGATCCATGAAGGTCCAGGGGTC-3’
**XhoI-BORF1-R**	5’-CCGCTCGAGCTAGAGAATCACCTC-3’
**BORF1-K196RK202R-F**	5’-GAGAATATAAGGAGCACCTATCTGAATAGAATCACCAC-3’
**BORF1-K196RK202R-R**	5’-GTGGTGATTCTATTCAGATAGGTGCTCCTTATATTCTC-3’
**BORF1-K2R-F**	5’-CGCGGATCCATGAGGGTCCAGGGGTCC-3’
**BORF1-K214RK222R-F**	5’-GTGGTCAGCAGGGCCATCCCTCGCAGCACCGTCAGGGTGACGGTG-3’
**BORF1-K214RK222R-R**	5’-CACCGTCACCCTGACGGTGCTGCGAGGGATGGCCCTGCTGACCAC-3’
**Ub-F-EcoRI**	5’-CCGGAATTCATGCAGATCTTCGTCAAGAC-3’
**Ub-R-HindIII**	5’-CCCAAGCTTTCAACCACCTCTTAGTCTTA-3’
**Ub-K48R-F**	5’-CTTTGCCGGTAGGCAGCTCGAG
**Ub-K48R-R**	5’- CTCGAGCTGCCTACCGGCAAAG
**UbK63R-R-HindIII**	5’-CCCAAGCTTTCAACCACCTCTTAGTCTTAAGACAAGATGTAAGGTCGACTCCCTC TGAATGTTG-3’
**UbK6RK11R-F-EcoRI**	5’-CCGGAATTCATGCAGATCTTCGTCAG GACGTTAACCGGTAGAACCATAAC-3’
**UbK27RK29R-F**	5’-GAAAACGTTAGGGCTAGGATTCAAGAC-3’
**UbK27RK29R-R**	5’-GTCTTGAATCCTAGCCCTAACGTTTTC-3’
**UbK33R-F**	5’-ATTCAAGACAGGGAAGGCATTC-3’
**UbK33R-R**	5’-GAATGCCTTCCCTGTCTTGAAT-3’
**UbK48-F**	5’-CTTTGCCGGTAAGCAGCTCGAG-3’
**UbK48-R**	5’-CTCGAGCTGCTTACCGGCAAAG-3’
**UbK63-R-HindIII**	5’-CCCAAGCTTTCAACCACCTCTTAGTCTTAAGACAAGATGTAAGGTCGACTCCTTCT GAATGTTG-3’
